# Elevated *ITGA2* expression promotes collagen type I-induced clonogenic growth of intrahepatic cholangiocarcinoma

**DOI:** 10.1038/s41598-022-26747-1

**Published:** 2022-12-27

**Authors:** Chotirat Rattanasinchai, Panida Navasumrit, Mathuros Ruchirawat

**Affiliations:** 1 Laboratory of Environmental Toxicology, Chulabhorn Research Institute, Bangkok, 10210 Thailand; 2 Center of Excellence on Environmental Health and Toxicology (EHT), OPS, Ministry of Higher Education, Science, Research and Innovation, Bangkok, Thailand

**Keywords:** Biochemistry, Cancer, Cell biology, Molecular biology

## Abstract

Intrahepatic cholangiocarcinoma (iCCA) arises along the peripheral bile ducts and is often accompanied by a tumor microenvironment (TME) high in extracellular matrices (ECMs). In this study, we aimed to evaluate whether an ECM-rich TME favors iCCA progression. We identified *ITGA2,* which encodes collagen-binding integrin α2, to be differentially-expressed in iCCA tumors compared with adjacent normal tissues. Elevated *ITGA2* is also positively-correlated with its ligand, collagen type I. Increased *ITGA2* expression and its role in collagen type I binding was validated in vitro using four iCCA cell lines, compared with a non-cancerous, cholangiocyte cell line. Robust interaction of iCCA cells with collagen type I was abolished by either *ITGA2* depletion or integrin α2β1-selective inhibitor treatment. In a phenotypic study, collagen type I significantly enhances clonogenic growth of HuCCA-1 and HuCCT-1 cells by three and sixfold, respectively. Inhibition of integrin α2 expression or its activity significantly blocks collagen type I-induced colony growth in both cell lines. Taken together, our data provide mechanistic evidence that collagen type I promotes growth of iCCA colonies through integrin α2 suggesting that the collagen type I—integrin α2 axis could be a promising target for cancer prevention and a therapeutic opportunity for this cancer.

## Introduction

Cholangiocarcinoma (CCA) is a rare but deadly cancer of the bile duct epithelium that can be classified into three subtypes: extrahepatic cholangiocarcinoma (eCCA), perihilar cholangiocarcinoma (pCCA) and intrahepatic cholangiocarcinoma (iCCA), depending on the location of the primary tumor^1^. In particular, iCCA, which arises along the peripheral bile ducts, has been a major health concern in many Asian countries^1,2^ while its incidence has also increased in western countries^3^. A major contributor to its lethality is the lack of effective therapies^4^. Surgical resection remains the only curative treatment for iCCA patients with localized disease. Still, only a small subset of patients benefit from this treatment due to late diagnosis^5^. Systemic chemotherapy remains largely ineffective. Therapeutic resistance is often encountered and in many cases the toxicity outweighs its benefit. As a consequence, most iCCA patients die within a year after diagnosis^6^.

Reciprocal interactions between cancer cells and the tumor microenvironment (TME) play a crucial role in all stages of cancer development, progression and therapeutic resistance^7–16^. iCCA is often surrounded with a desmoplastic TME as a consequence of chronic inflammation in the bile duct epithelium and nearby tissues^17^. This reactive stroma is high in tumor promoting cells which continuously remodel the extracellular matrix (ECM) composition^18^ leading to the accumulation of fibrillar collagen type I^19^. In many cancers, a collagen type I-rich TME has been shown to promote cancer aggressiveness and is predictive of a poor outcome^20^.

ECM signals are transduced into cells through various cell surface receptors; among them are integrin members. These transmembrane alpha (α) beta (β) integrin heterodimers serve as master regulators of cell–matrix interaction that bind directly to specific ECMs and transduce the signals into intracellular components^21^. Aberrant expression of integrin members is commonly found in a variety of cancers where they function to promote cancer progression and metastasis through different downstream signaling pathways^22^. Of the 18 α-subunits and 8 β-subunits, four integrin heterodimers: integrin α1β1, α2β1, α10β1 and α11β1, are identified as collagen receptors^23^.

Herein, we demonstrate that integrin α2 is highly upregulated in iCCA cells and serves as a key mediator in iCCA-collagen type I interaction. Disrupting integrin α2 activity by either gene silencing or inhibitor treatment blocked collagen type I-induced growth of iCCA colonies in vitro. Our data suggest that high integrin α2 expression in iCCA cells facilitates cancer progression in a collagen type I-rich TME.

## Results

### Aberrant upregulation of *ITGA2* in iCCA tumors.

**Figure 1:**
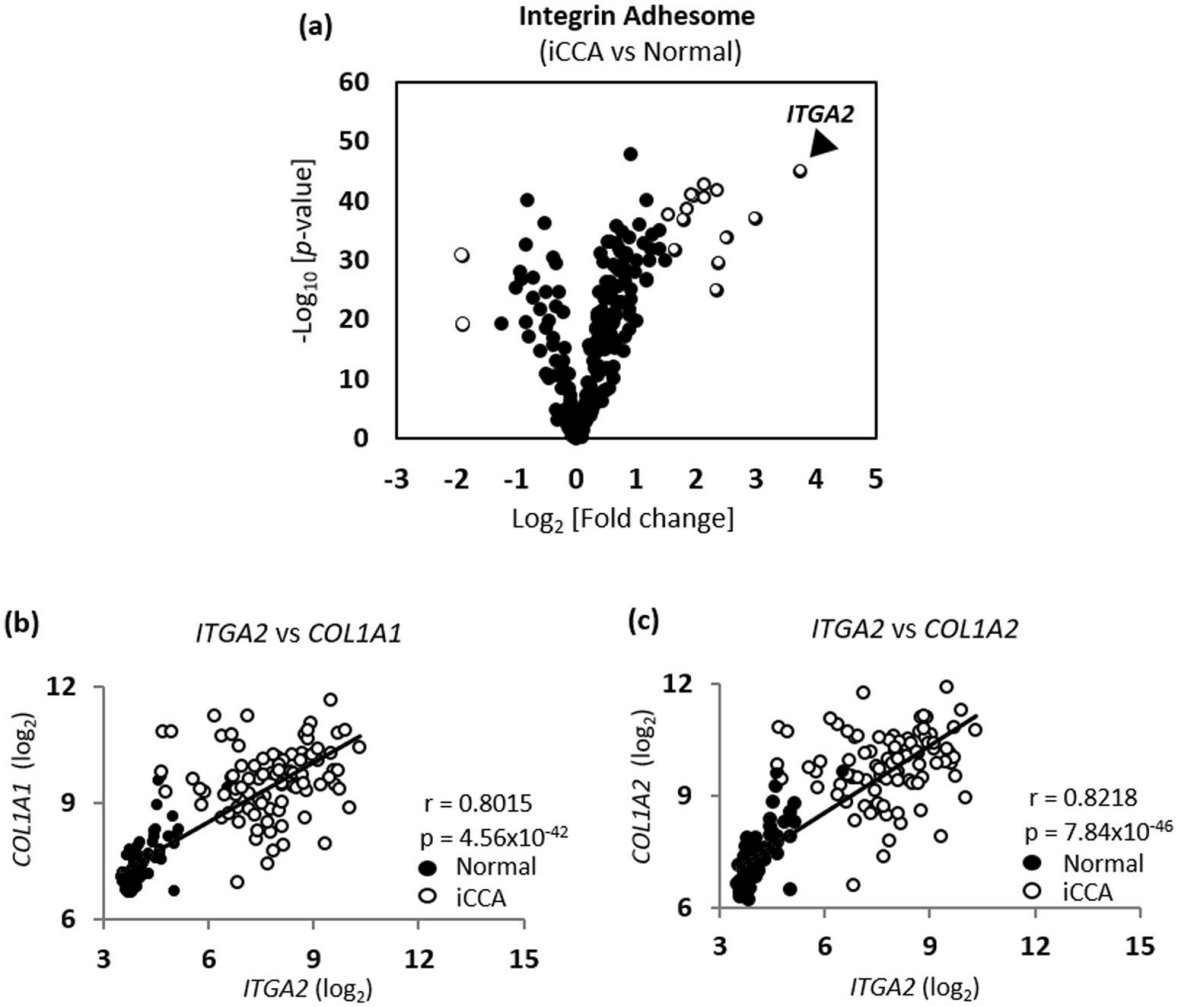
Aberrant *ITGA2* upregulation is correlated with high collagen type I expression in iCCA tumors. (**a**) Volcano plot of the differentially-expressed integrin adhesome-related genes between iCCA tumors and their adjacent normal tissues from GSE76297 dataset (n = 91). Black dots indicate genes with no or low differential expression. White dots indicate genes with high differential expression (Log_2_FC ≥ 1.5 or Log_2_FC ≤ − 1.5). Co-expression of genes between either (**b**) *ITGA2* and *COL1A1,* or (**c**) *ITGA2* and *COL1A2* from the same dataset were analyzed using Pearson’s r correlation.

Desmoplastic stroma high in collagen type I is among key characteristics of CCA^18^. To elucidate the interplay between collagen type I-rich TME and iCCA progression, we first examined the gene expression profile of the integrin adhesome network^24^, which consists of integrins, major cell–matrix adhesion receptors, and their downstream signaling effectors on a GEO dataset, GSE76297, of Thai iCCA samples and their adjacent normal tissue counterparts (n = 91)^25^. A total of 232 genes from the integrin adhesome network were analyzed using paired t-test analysis to determine the differential gene expression between iCCA samples and their normal tissue counterparts (Fig. 1a). With a cutoff of Log_2_FC ≥ 1.5 or Log_2_FC ≤ − 1.5, a total of 16 integrin adhesome-related genes were found differentially-expressed in iCCA tumors compared with their adjacent normal tissues (Supplementary Fig. 1a). Among this gene list, *ITGA2,* which encodes integrin α2, was the most upregulated gene in iCCA samples with 13-fold increased expression (*p*-value = 1.03 × 10^−45^) compared with their normal tissue counterparts. Given that integrin α2 forms a heterodimeric complex with integrin β1 to function as a collagen receptor, we hypothesized that elevated integrin α2, coupling with integrin β1, in iCCA cells could be crucial for iCCA progression in collagen type I-rich TME through direct cell–matrix adhesion.

To further support this hypothesis, we performed co-expression analyses of integrin α2 and two highly-expressed forms of collagen type I, *COL1A1* and *COL1A2*, on the same GEO dataset (GSE76297). As shown in Fig. 1b,c, the expression levels of *ITGA2* and two isoforms of genes encoding collagen type I (*CO1A1* and *COL1A2*) were remarkably higher in iCCA samples (white dots) when compared with the normal tissues (black dots). In addition, the *ITGA2* expression level was positively correlated with both isoforms of collagen type I with a Pearson’s r of 0.8015 and 0.8218 for *COL1A1* and *COL1A2*, respectively.

### Intrahepatic cholangiocarcinoma cells exhibit high basal integrin α2 expression, recapitulating iCCA tumors

**Figure 2:**
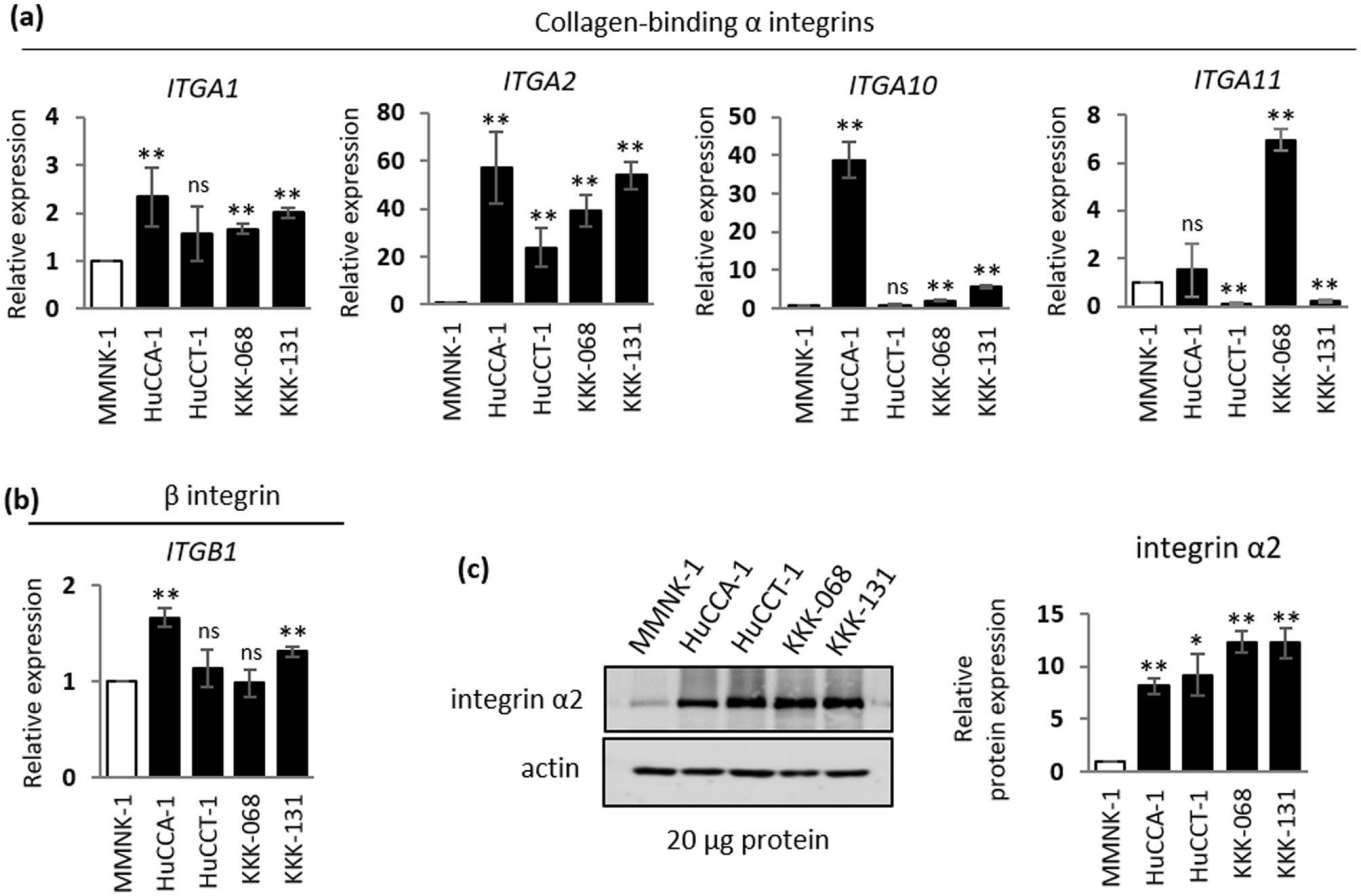
Intrahepatic cholangiocarcinoma cells display high basal integrin α2 expression. Relative gene expression of (**a**) collagen-binding integrin α subunits: *ITGA1, ITGA2, ITGA10* and *ITGA11*, and their partner (**b**) integrin β subunit, *ITGB1,* from a non-cancerous MMNK-1 cholangiocyte cell line or four iCCA cell lines: HuCCA-1, HuCCT-1, KKK-068 and KKK-131. (**c**) Immunoblot analysis of integrin α2 protein expression in MMNK-1 cells and four iCCA cell lines, actin serves as an internal control. Quantification from three independent experiments are shown on the right panel; white bars, a non-cancerous cell line; black bars, cancerous cell lines. All data are expressed as the mean ± SD from at least three independent experiments. The Student’s t-test was used to calculate *p*-value, compared with MMNK-1 control. ns, not significant; *p < 0.05; **p < 0.01.

Because gene expression data from iCCA tumors indicated the potential involvement of integrin α2 in iCCA progression in the collagen type I-rich TME, we investigated the underlying mechanism of how collagen type I exerts its effect on iCCA progression by using iCCA cell models. To determine whether the collagen type I-specific integrin expression pattern in iCCA cell lines recapitulates that found in the iCCA tumors, gene expression analysis of four α subunits (*ITGA1*, *ITGA2*, *ITGA10*, and *ITGA11*) and one β subunit (*ITGB1*) were assessed in four iCCA cell lines (HuCCA-1^26^, HuCCT-1^27^, KKK-068^28^ and KKK-131^28^) and compared with that in the non-cancerous, cholangiocyte cell line, MMNK-1^29^. As shown in Fig. 2a, all iCCA cell lines tested displayed significantly elevated *ITGA2* transcript levels with 57, 24, 39 and 54-fold increases in HuCCA-1, HuCCT-1, KKK-068 and KKK-131 cells, respectively, when compared with MMNK-1 cells. Although only small or inconsistent transcript levels of other integrin subunits was observed in this experiment, the trends were consistent with those found in human data sets of iCCA tumors (Fig. 2a,b and Supplementary Fig. 1b). Increased *ITGA2* expression was also confirmed at the protein level using immunoblotting. Consistent with the gene expression data, all four iCCA cell lines exhibited high levels of integrin α2 when compared with the level in cholangiocyte MMNK-1 cells (Fig. 2c, original blots are presented in Supplementary Fig. 2).

### Intrahepatic cholangiocarcinoma cell lines display strong cell adhesion to collagen type I, and this interaction is mediated by integrin α2

**Figure 3:**
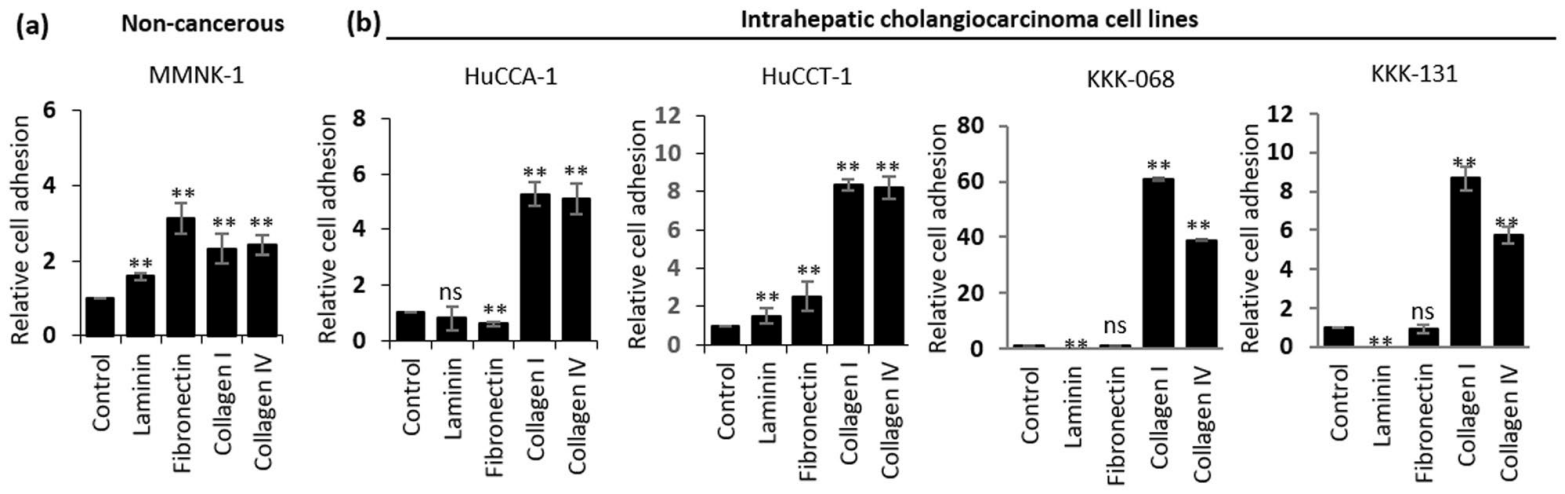
Intrahepatic cholangiocarcinoma cells display strong adhesion to collagen type I. (**a**) MMNK-1 cells or (**b**) four cancerous iCCA cell lines (HuCCA-1, HuCCT-1, KKK-068 and KKK-131) in a cell adhesion assay on uncoated control or indicated extracellular matrices at concentrations of 10 µg/mL for 1 h. The bar graphs represent the mean ± SD from at least three independent experiments. The Student’s t-test was used to calculate *p*-value, compared with a control group. ns, not significant; *p < 0.05; **p < 0.01.

Integrin α2 heterodimerized with integrin β1 recognizes and binds with high affinity to collagen type I and, to some extent, collagen type IV^23^. Given that iCCA cells express high levels of integrin α2, these cells could possibly favor binding to collagen fibers, particularly collagen type I. To assess the direct cell–matrix interaction, cell adhesion assays were utilized to determine the binding capacity of iCCA cells to collagen type I compared with other matrices including: laminin, fibronectin and collagen type IV. As shown in Fig. 3a, cholangiocyte MMNK-1 cells were able to adhere to all matrices tested with the highest binding capacity to fibronectin, consistent with the normal physiological condition where fibronectin is a major protein in the biliary basement membrane^30^. Interestingly, all four iCCA cell lines tested in this experiment displayed strong adhesion to collagen type I and IV (Fig. 3b).

**Figure 4:**
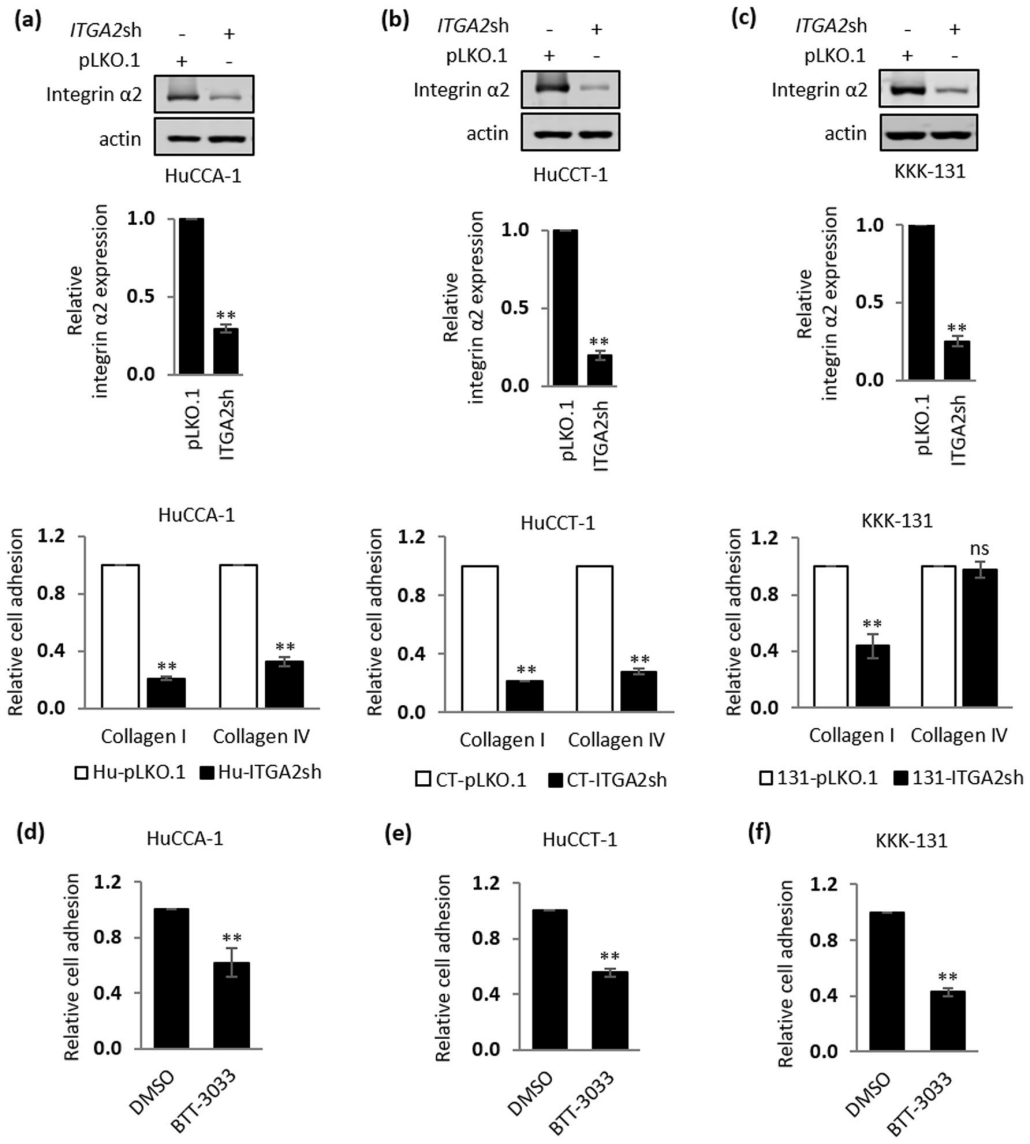
Integrin α2 is critical for a direct interaction between iCCA cells and collagen type I. (**a**) HuCCA-1, (**b**) HuCCT-1 and (**c**) KKK-131 cells stably expressing pLKO.1 vector control or pLKO.1-*ITGA2*shRNA (*ITGA2*sh) were subjected to a cell adhesion assay on 10 μg/mL collagen type I or IV for 1 h. Top: Representative immunoblot analysis against integrin α2. Actin serves as a loading control. Middle: Quantification of immunoblot analysis against integrin α2. Bottom: Quantification of cell adhesion assays. (**d**) HuCCA-1, (**e**) HuCCT-1 and (**f**) KKK-131 cells ± DMSO or 5 μM BTT-3033 (an integrin α2β1 selective inhibitor) were subjected to 1 h adhesion assay. The bar graphs represent the mean ± SD from at least three independent experiments. The Student’s t-test was used to calculate *p*-value, compared with a control group. ns, not significant; **p < 0.01.

To test whether integrin α2 is required for iCCA-collagen type I adhesion, iCCA cells were first engineered to express either pLKO.1 as an empty vector control or pLKO.1-*ITGA2*sh expressing an *ITGA2*-specific shRNA. Notably, only three engineered cell lines: HuCCA-1, HuCCT-1 and KKK-131, were successfully established. As shown in Fig. 4a–c (Top and Middle panel, original blots are presented in Supplementary Fig. 3a,b), reduction of integrin α2 protein expression by 60–80% was observed in HuCCA-1, HuCCT-1, and KKK131 stably expressing *ITGA2*shRNA when compared with the corresponding cells stably expressing pLKO.1 vector control. In addition, these *ITGA2* stably-silenced iCCA cells displayed a decrease in cell-collagen type I interaction when compared with iCCA cells stably expressing the empty vector control (Fig. 4a–c, bottom panel). Notably, the role of integrin α2 in mediating iCCA cell-collagen type IV interaction appears to be cell-type specific as only *ITGA2* stably-silenced HuCCA-1 and HuCCT-1 cells but not KKK-131 cells showed decreased cell adhesion to collagen type IV (Fig. 4c, bottom panel), possibly though the expression of other collagen type IV receptors in KKK-131 cells. In order to confirm the specificity of the *ITGA2* knockdown cell adhesion phenotype, transient knockdown of integrin α2 was also conducted in HuCCA-1 cells using two different siRNAs targeting distinct regions of the *ITGA2* mRNA (si*ITGA2*#1 and si*ITGA2*#2). These cells were subsequently subjected to cell adhesion assays**.** Immunoblot analysis against integrin α2 in HuCCA-1 cells treated with the two si*ITGA2* sequences showed 70–80% protein knockdown efficiency when compared with cells treated with a scrambled control siRNA (siCON) (Supplementary Fig. 4a). Consistent with the results observed in *ITGA2* stably-silenced HuCCA-1 cells, transient integrin α2 silencing in HuCCA-1 cells decreased their ability to bind to both collagen type I and IV (Supplementary Fig. 4b). Furthermore, treatment with BTT-3033, an integrin α2β1 selective inhibitor, is also sufficient to decrease the binding of HuCCA-1, HuCCT-1, and KKK-131 cells to collagen type I (Fig. 4d–f). Taken together, these data strongly suggest that integrin α2 is a major molecule mediating the direct interaction between iCCA cells and collagen type I. Thus, collagen type I may directly exert its influence on iCCA cells through this interaction.

### Collagen type I promotes a clonogenic growth of iCCA cells

**Figure 5:**
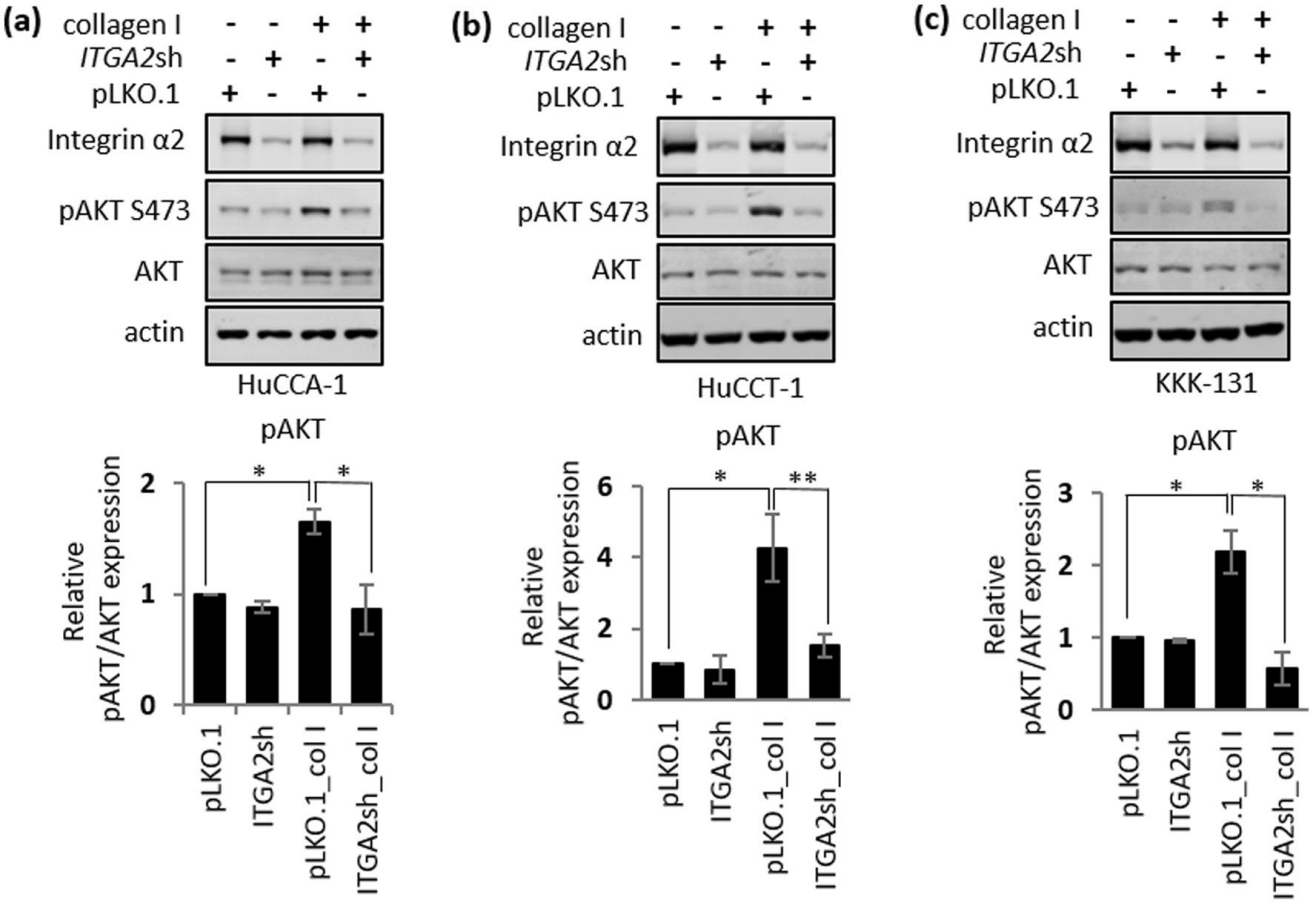
Integrin α2 is required for collagen type I-mediated AKT phosphorylation. Immunoblot analysis of (**a**) HuCCA-1, (**b**) HuCCT-1, and **(c)** KKK-131 cells stably expressing pLKO.1 vector control or pLKO.1-*ITGA2*shRNA (*ITGA2*sh) after 1 h adhesion on uncoated or 10 μg/mL collagen I. Actin serves as a loading control. Quantification of band intensity was shown on the bottom. Relative phosphorylated protein expressions were normalized to the corresponding total protein. The bar graphs represent the mean ± SD from at least three independent experiments. The Student’s t-test was used to calculate *p*-value. *p < 0.05; **p < 0.01.

To investigate how collagen type I provides beneficial TME to iCCA cells, we first evaluated the major signaling pathway(s) that could be influenced by collagen type I in iCCA cells. As shown in Fig. 5a–c (original blots are presented in Supplementary Fig. 5a–c), collagen type I is sufficient to induce the activation of AKT, a signaling molecule that plays critical roles in cancer cell growth and survival^31^; this activation, as evidenced by the phosphorylation of AKT at serine 473, is inhibited in *ITGA2* stably silencing iCCA cells. Altogether, our data demonstrated that *ITGA2* is a critical mediator for collagen type I-induced AKT activation suggesting that this collagen type I-*ITGA2* axis could promote growth and survival of iCCA.

**Figure 6:**
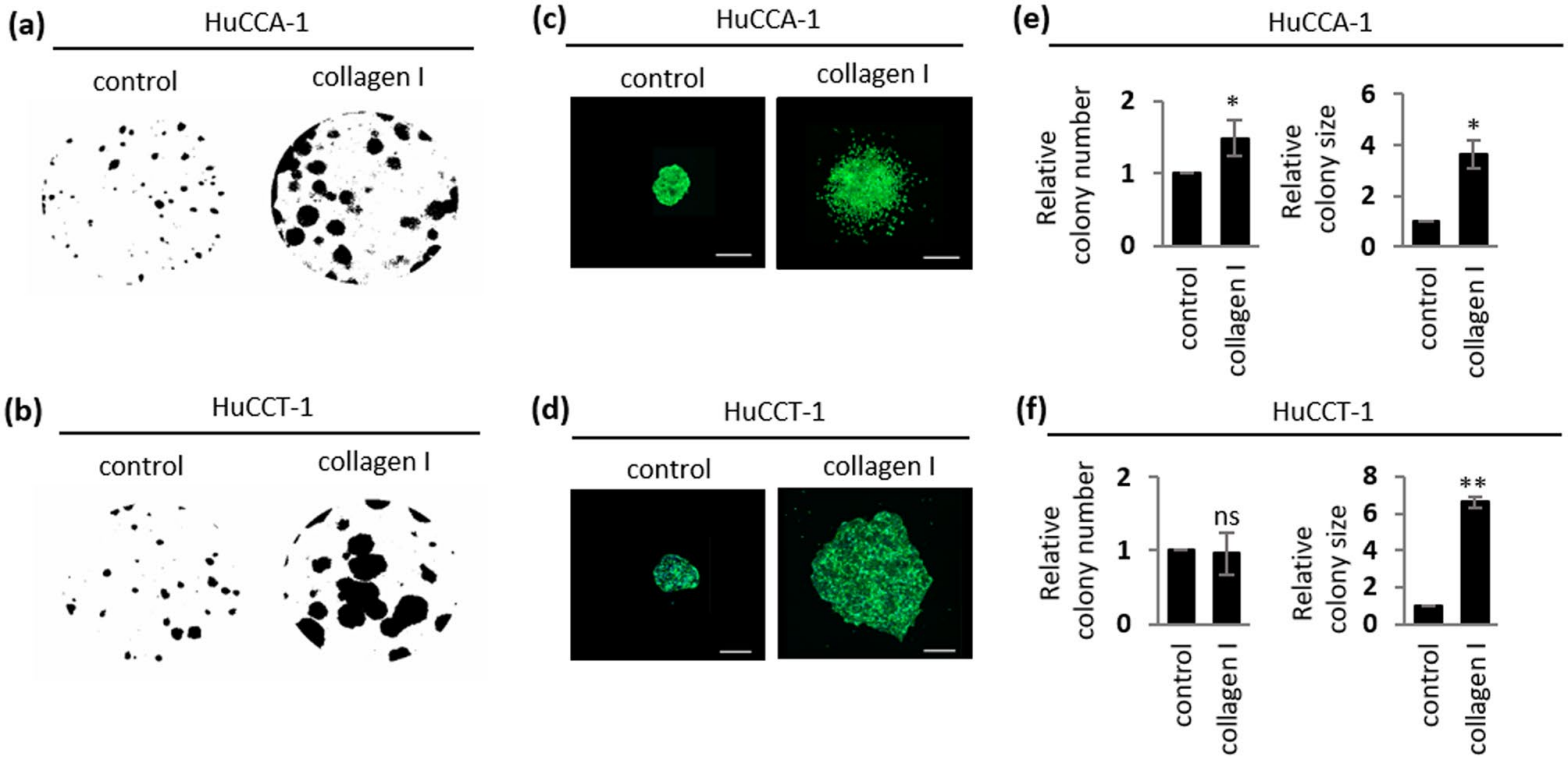
Collagen type I promotes clonogenic growth of iCCA cells. Parental HuCCA-1 and HuCCT-1 ± 10 μg/mL collagen type I in a 10-day clonogenic assay; (**a,b**) whole well images; (**c,d**) Alexa Fluor 488 Phalloidin-stained HuCCA-1 and HuCCT-1 colonies, Scale bar = 500 μm and (**e,f**) quantifications of colony number and size. All bar graphs represent the mean ± SD from at least three independent experiments. The Student’s t-test was used to calculate *p*-value. ns, not significant; *p < 0.05; **p < 0.01.

To investigate the impact of collagen type I on iCCA growth and survival, an in vitro clonogenic assay was chosen to determine the ability of individual cells to survive in a stressful environment and proliferate to form multicellular colonies^32,33^. Considering all three iCCA cell lines exhibit a similar AKT activation upon a collagen type I treatment (Fig. 5a–c), we have selected HuCCA-1 and HuCCT-1, the two most widely used iCCA cell lines out of all three cell lines, to test for their clonogenicity in the presence or absence of collagen type I. As shown in Fig. 6a,b, several HuCCA-1 and HuCCT-1 cells were able to survive and form multicellular colonies within 7–10 days. In addition, the colonies of HuCCA-1 and HuCCT-1 grown in the absence of collagen type I exhibited a round, smooth-edge morphology while the colonies grown in the presence of collagen type I were larger and displayed invasive characteristics as indicated by increased disseminating cells around the colonies (Fig. 6c,d). Although collagen type I had a small or negligible effect on the number of colonies which represent the number of surviving cells, it significantly enhanced the clonogenic growth of iCCA cells as judged by increased colony size by approximately 3 and sixfold in HuCCA-1 and HuCCT-1, respectively, compared to their control counterparts (Fig. 6e,f).

**Figure 7:**
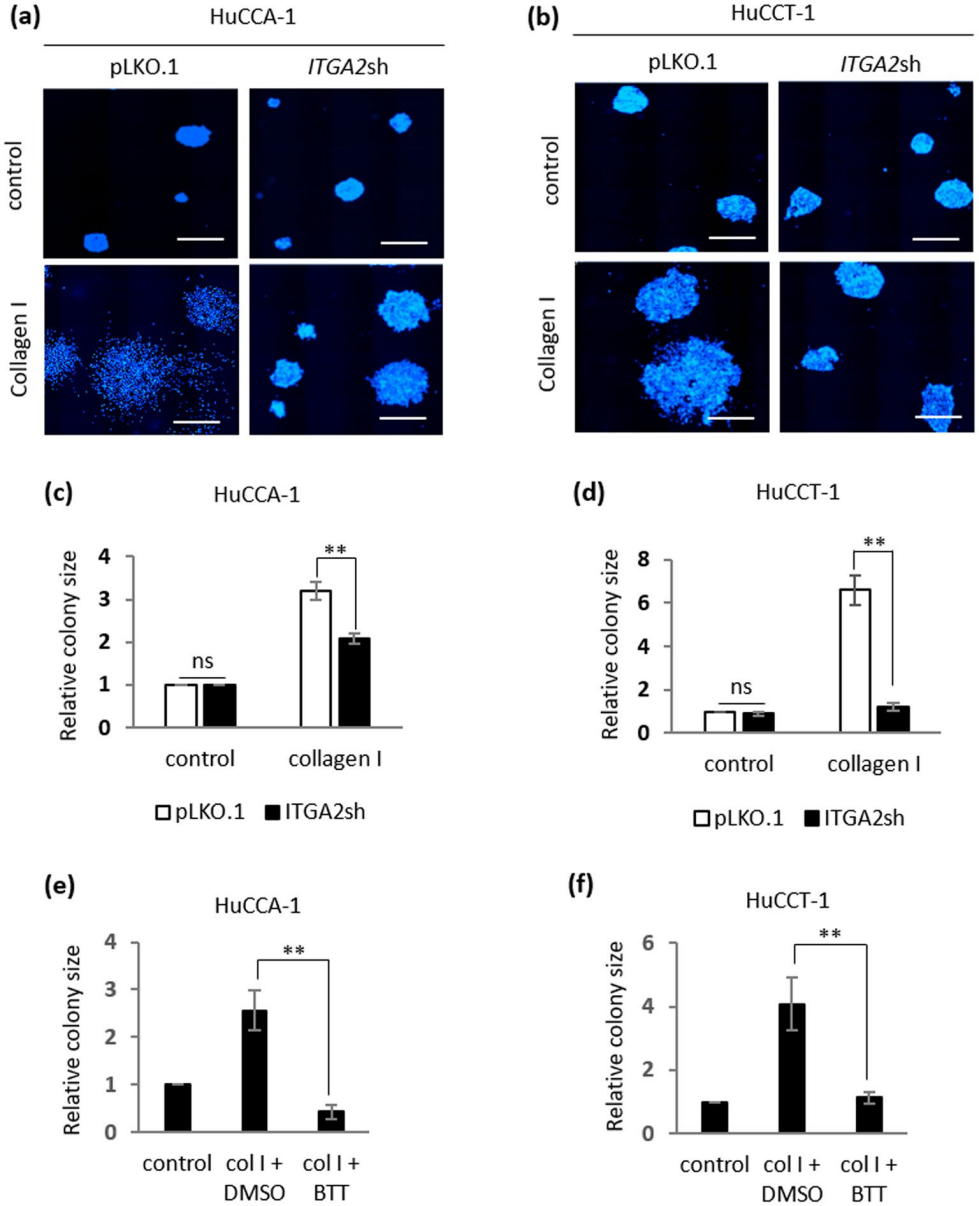
Interaction between collagen type I and integrin α2 is necessary for collagen type I-induced clonogenic growth of iCCA cells. HuCCA-1 and HuCCT-1 cells stably expressing pLKO.1 vector control or pLKO.1-*ITGA2*sh ± 10 μg/mL collagen type I in clonogenic assays for up to 10 days. (**a,b**) DAPI-stained HuCCA-1 and HuCCT-1 colonies. Scale bar = 1000 μm. Quantification of colony size from (**c**) HuCCA-1 ± pLKO.1 vs. HuCCA-1 ± pLKO.1-*ITGA2*sh and (**d**) HuCCT-1 ± pLKO.1 vs. HuCCT-1 ± pLKO.1-*ITGA2*sh. Quantification of (**e**) HuCCA-1 ± DMSO or BTT-3033 (5 μM) and (**f**) HuCCT-1 ± DMSO or BTT-3033 (5 μM) in the presence or absence of 10 μg/mL collagen type I. All bar graphs represent the mean ± SD from at least three independent experiments. The Student’s t-test was used to calculate *p*-value. ns, not significant; **p < 0.01.

To test whether this collagen type I-induced, clonogenic growth is mediated by integrin α2, HuCCA-1 and HuCCT-1 cells stably expressing pLKO.1 vector control and pLKO.1-*ITGA2*sh were subjected to a clonogenic assay in the presence of collagen type I. In uncoated control, no difference in colony size was observed in HuCCA-1 or HuCCT-1 stably expressing pLKO.1 vector control and pLKO.1-*ITGA2*sh (Fig. 7a,b). Similar to parental cells, both HuCCA-1 and HuCCT-1 expressing vector control exhibit increased colony size. Stably *ITGA2* silencing significantly impaired collagen type I-induced, clonogenic growth of both HuCCA-1 and HuCCT-1 cells (Fig. 7c,d), consistent with the result from the cell proliferation assay demonstrating that *ITGA2*-mediated collagen type I signals are sufficient to induce iCCA cell proliferation (Supplementary Fig. 6). In addition to a decrease in colony size, the disseminating cells, which reflect the invasive character of these iCCA cells, were also absent in *ITGA2* silencing cells. The requirement of integrin α2 in mediating collagen type I signaling was further confirmed by treatment with the integrin α2β1 inhibitor, BTT-3033. As shown in Fig. 7e,f, treatment with BTT-3033 reversed the effect of collagen type I-induced clonogenic growth of both HuCCA-1 and HuCCT-1 cells. Taken together, our results demonstrate that, upon binding to collagen type I, integrin α2 functions to transduce collagen type I signal to promote growth of iCCA cells in clonogenic assays.

## Discussion

While risk factors for CCA vary geographically^4^, the disease usually involves chronically-inflamed biliary epithelia and a subsequent desmoplastic reaction with the formation of dense collagen fibers^34,35^. This unique TME is critical for cancer progression^36^ by providing environmental cues to dictate what oncogenic signaling thrives within the tumors. Nevertheless, CCA tumors that arise on different locations of the biliary tree*, i.e*. iCCA and eCCA, still exhibit unique pathophysiological characteristics, possibly due to the different cells of origin. In the context of iCCA, we found several genes involved in integrin-mediated, cell-ECM adhesion to be differentially-expressed within iCCA tumors when compared with a non-cancerous cell line. Many integrin genes, *i.e. ITGA2*, *ITGA3, ITGA6*, *ITGB4, ITGB6*, and *ITGB8,* are upregulated within tumors, with increases in gene expression ranging from 3-fold (*ITGA3*) to 13-fold (*ITGA2*) (Supplementary Fig. 1a). Concomitantly, we found that the expression of *ITGA2*, the most upregulated integrin member among the list, to be positively correlated with its specific ligand, collagen type I, within the same dataset. Given the vast number of cell types within iCCA tumors, our gene expression analyses of iCCA cell lines strongly suggests that iCCA cells are responsible for increased *ITGA2* expression within the tumor mass.

Indeed, evidence of elevated *ITGA2* gene expression, regardless of tumor subtype or other etiological factors, has been previously observed in two cohorts of iCCA patients from two different ethnic groups^37,38^. Though no molecular mechanism has been proposed, *ITGA2* expression predicted a poor prognosis for iCCA patients in both studies. Mechanistically, we describe here that iCCA cells bind strongly to collagen type I matrices via a direct interaction with integrin α2 and this interaction is necessary to promote cell growth and confer invasive characteristics to iCCA colonies in clonogenic assays. Both gene silencing and inhibitor treatment approaches were utilized here to confirm that either loss of integrin α2 expression or inhibition of its ligand binding ability is sufficient to reduce the effect of collagen type I on iCCA cells. These data suggest that integrin α2 may provide a growth benefit to iCCA tumors in the presence of high collagen type I in the TME.

Critical roles of integrin α2 in cancer growth and progression have also been recognized in other cancer types^39^ including: glioblastoma^40^, prostate cancer^41^, pancreatic cancer^42–44^ and gastric cancer^45^. In prostate cancer, the elevated expression of integrin α2 is associated with tumor aggressiveness^46^. Notably, high integrin α2β1 serves as a biomarker for prostate progenitor cells^39^. Its expression was required for tumor-initiating cells in a prostate cancer xenograft model and this tumorigenicity could be predicted using an in vitro clonogenic assay^47^. Indeed, our results from clonogenic assays also suggest the potential involvement of *ITGA2* in the establishment of stemness characteristics of iCCA in the TME high in collagen type I. Likewise, the role of *ITGA2* in tumor-initiating cells has also been described in a triple-negative breast cancer model^48^. Recently, three research groups have identified *ITGA2* as a potential therapeutic target for treating pancreatic cancer^42–44^, another cancer type that shares the characteristic of desmoplastic stroma high in collagen type I^49,50^. Our data, along with the role of *ITGA2* in other cancers, suggest that *ITGA2* signaling can be a potential molecular target for therapeutic development and a predictive biomarker for iCCA progression.

Tumorigenic ECM remodeling is a dynamic process shaped by cancer cells and their supporting stromal and immune cells^7,51^. This remodeling, which involves ECM deposition, ECM degradation and changes in ECM organization, alters ECM architecture around solid tumors^51^, and, in turn, provides biochemical and mechanical cues to cancer cells ^52^. In many cancers, the accumulation of collagen type I is commonly found and leads to increased integrin-mediated, epithelial-to-mesenchymal transition (EMT) driving migration and invasion of cancer cells to surrounding tissues^20,52–55^. In this study, we observed that collagen type I enhanced cell dissemination around HuCCA-1 and HuCCT-1 colonies, which is abrogated by *ITGA2*-silencing in iCCA cells. Because this phenotype partly reflects EMT, it is possible that the collagen type I-integrin α2 axis orchestrates the expression of a subset of genes to promote EMT in iCCA cells. Increased numbers of collagen receptors on the cell surface also links to forced-mediated ECM modification that causes the alignment of collagen fibers to support growth and invasion of cancer cells^51^. Indeed, *ITGA2* 1648G > A polymorphism which causes integrin α2 to be densely localized on the cell surface^39^ is associated with increased risk of developing breast cancer^56^. Although we did not directly prove whether integrin α2 clusters were present on the surface of iCCA cells, the ability of these cells to strongly bind to a collagen type I matrix combined with the observation that binding is abrogated by either *ITGA2* gene silencing or treatment with an integrin α2β1 selective inhibitor strongly indicates the existence of integrin α2 clusters on the iCCA cell surface. Further investigations focusing on how integrin α2 on the surface of iCCA cells remodels the surrounding collagen fibers to their advantage would provide further insight into the role of this protein in iCCA progression.

In summary, we provide evidence that high integrin α2 co-exists with high collagen type I in iCCA tumors. Mechanistically, this receptor-ligand interaction provides a growth benefit for iCCA colonies in vitro. As a consequence, disruption of this axis by either *ITGA2* depletion or inhibition of receptor-ligand interaction blocks both collagen type I-induced iCCA growth and development of invasive characteristics. Our results indicate that high integrin α2 expression provides advantages for iCCA growth and progression in a TME high in collagen type I.

## Materials and methods

### Extracellular matrices, chemicals and antibodies

#### Extracellular matrices

Rat tail collagen type I (#354236), mouse laminin (#354232), mouse collagen type IV (#354233), and human fibronectin (#356008) were from Corning.

#### Chemicals

Selective Integrin α2β1 inhibitor: BTT 3033 was from Tocris Bioscience (Bristol, UK). Bovine serum albumin (BSA), 4′,6-Diamidino-2-phenylindole dihydrochloride (DAPI) and 3-(4,5-Dimethyl-2-thiazolyl)-2,5-diphenyl-2H-tetrazolium bromide (MTT) were purchased from Sigma-Aldrich. All reagents for cell culture were obtained from Invitrogen (Thermo Fisher) except fetal bovine serum (FBS), which was obtained from Millipore. Alexa Fluor 488 Phalloidin was supplied by Cell Signaling.

#### Antibodies

Anti-β-actin (clone C4, #SC-47778) was obtained from Santa Cruz Biotechnology. Anti-integrin α2 (CD49b, #ab1936) was supplied by Abcam. Anti-phospho-AKT (S473) (D9E, #4060) and anti-pan-AKT (40D4, #2920) were purchased from Cell Signaling. IRdye 800CW goat anti-mouse IgG, IRdye 680 goat anti-rabbit IgG were obtained from Li-Cor Bioscience.

### Cell lines

Cholangiocyte MMNK-1 and two iCCA cell lines: KKK-068 and KKK-131, obtained from JCRB cell bank, Japan, were maintained in DMEM supplemented with 5% fetal bovine serum (FBS) (MMNK-1) or 10% FBS (KKK-068 and KKK-131), 1% GlutaMAX, and 1% Penicillin/streptomycin (P/S). HuCCA-1 iCCA cells, described previously^57^, were maintained in Ham F-12 supplemented with 10% FBS, 1% GlutaMAX, and 1% P/S. HuCCT-1 iCCA cells obtained from RIKEN, Japan, were maintained in RPMI-1640 supplemented with 10% FBS and 1% P/S. All cell lines were grown at 37 °C under a 5% CO_2_ atmosphere and were routinely tested for mycoplasma contamination using DAPI staining. All cell lines, except HuCCA-1, were recently purchased. HuCCA-1 cells were recently authenticated at CLS cell lines service, Germany.

### Transient and stable ITGA2 silencing

#### Reverse transfection for siRNA-mediated transient gene silencing

Two pre-designed siRNA duplexes targeting human *ITGA2* (NM_002203): si*ITGA2*#1 (#SASI_Hs01_0012382, sense 5’-CGAAAGUAAUGGUAGUUGU-3’ and anti-sense 5’-ACAACUACCAUUACUUUCG-3’) and si*ITGA2*#2 (#SASI_Hs01_0012383, sense 5’ –CUGGUUACUGGUUGGUUCA-3’and anti-sense 5’-UGAACCAACCAGUAACCAG-3’) were obtained from Sigma-Aldrich. Ambion Silencer Select Negative control #1 siRNA was supplied by Thermo Fisher. Lipofectamine 2000 (Invitrogen) was used as a transfection reagent and reverse transfection was performed according to the manufacturer’s protocol. A final concentration of 100 nM siRNA was used in all experiments. After at least 40 h incubation, the cells were harvested for further analysis.

#### Lentiviral transduction for shRNA-mediated stable gene silencing

Lentiviral particles containing either human *ITGA2*shRNA (5’-AGGTAAACTAACCTGGTATTT-3) or control vector (pLKO.1-puro) were purchased from Sigma-Aldrich. Cells were infected with lentiviral particles according to the manufacturer’s protocol. Polybrene was added during lentiviral infection at a final concentration of 4 μg/mL. After 72 h, puromycin was added into the cell culture at a final concentration of 2 μg/mL for HuCCA-1 cells, and 1 μg/mL for HuCCT-1 and KKK-131. After a two-week selection, all cells were maintained in growth medium supplemented with 1 μg/mL puromycin.

### Quantitative real-time PCR

Total RNAs were extracted using an RNeasy Mini kit (Qiagen) and subsequently subjected to cDNA synthesis using qScript cDNA Supermix (Quanta bio), according to the manufacturers’ protocols. Real-time qPCR was performed using Thunderbird SyBR qPCR Mix (Toyobo, Japan) and a QuantStudio 3 Real-Time PCR system (Applied Biosystems, Thermo Fisher). Specific primer sequences^58,59^ used in this study are shown in Table 1.

**Table 1:** List of primers.

Gene	Forward primer (5' to 3')	Reverse primer (5' to 3')	References
*ITGA1*	GCTCCTCACTGTTGTTCTACG	CGGGCCGCTGAAAGTCATT	PrimerBank^58^: ID 31657141c1
*ITGA2*	GGGAATCAGTATTACACAACGGG	CCACAACATCTATGAGGGAAGGG	PrimerBank^58^: ID 116295257c2
*ITGA10*	GGTGGGACTGGTACAGTATGG	CACTTCTTCCTTCGTTCGGAAAT	PrimerBank^58^: ID 38569397c3
*ITGA11*	GTGGCAATAAGTGGCTGGTC	GTTCCCGTGGATCACTGGAC	PrimerBank^58^: ID 52485852c1
*ITGB1*	GTAACCAACCGTAGCAAAGGA	TCCCCTGATCTTAATCGCAAAAC	PrimerBank^58^: ID 182507160c2
*GAPDH*	GGCTGAGAACGGGAAGCTTG TCAT	AGCCTTCTCCATGGTGGTGAAGA	Rattanasinchai et al.^59^

### Cell adhesion assay

Cell adhesion assays were performed as previously described^60^. Briefly, 96-well plates were pre-coated for 1 h with 10 μM of the indicated matrices (collagen type I, fibronectin, laminin, or collagen type IV), followed by 30 min incubation with 0.2% BSA in phosphate buffered saline (PBS). Subsequently, 10,000 cells in 100 μL were introduced into each well. Cells were allowed to adhere for 1 h at 37 °C, under 5% CO_2_. Images were obtained using a phase contrast microscope. Plates were rinsed three times with PBS to eliminate unattached cells. The attached cells were then quantified using a 3-(4,5-dimethylthiazol-2-yl)-2,5-diphenyltetrazolium bromide (MTT) assay following the manufacturer’s protocol.

### Clonogenic assay

Clonogenic assays were performed as previously described^32^. Briefly, 100 cells in growth medium were introduced onto 6-well plates pre-coated with 10 μg/mL collagen type I. The colony formation was followed for up to 10 days. If indicated, BTT-3033, a selective α2β1 integrin inhibitor, was added in the cell suspension at the beginning of the experiment. The medium was replaced twice weekly and the inhibitor was freshly added if applied. At the end, colonies were stained with AlexaFluor 488-conjugated phalloidin to visualize cell bodies (actin cytoskeleton) and counterstained with DAPI for nuclear staining. Images were obtained using a Lionheart live cell imaging system (Biotek, Agilent Santa Clara, CA, USA) and analyzed with Gen 5 prime software (Biotek, Agilent Santa Clara, CA, USA) for the total size of the colonies. ImageJ software was used to convert fluorescence images into black-white images. At least three independent experiments done in duplicate were performed.

### Immunoblot analysis

Immunoblotting was performed as previously described^59^. Briefly, cell lysates were prepared in ice-cold lysis buffer (1% Triton X-100, 150 mM sodium chloride and 50 mM Tris, pH 8.0) freshly supplemented with phosphatase inhibitor cocktail 2 (Sigma) and protease inhibitor cocktail (Sigma). Protein lysates (15–30 µg) were subjected to gel electrophoresis, transferred to PVDF-FL membranes (Immobilon, Millipore). The membranes were blocked with 1 × Odyssey blocking buffer in PBS (Li-Cor), probed with the indicated primary antibodies (1:1000 or as indicated) and, subsequently, probed with secondary antibodies (1:5000). Image acquisition and data quantification were performed using an Odyssey CLx (Li-Cor) and Image Studio software.

### GEO data analysis

Gene expression data of iCCA tumors compared with their adjacent normal tissue counterpart was downloaded from the GEO database (GSE76297)^25^. The list of the integrin adhesome network was previously described^24^. Differential expression of integrin adhesomes from iCCA tumors and their normal adjacent tissues were analyzed using paired t-test analysis.

### Statistical analysis

Statistical analysis of GSE76297 dataset was performed using GenePattern (https://cloud.genepattern.org/gp)^61^. The results from in vitro data are expressed as the mean ± standard deviation (SD) from at least three independent experiments. The two-tailed Student’s t-test was used to calculate the *p*-value.

## Supplementary Information

Supplementary Figures.

## Data Availability

The dataset analyzed during this study is available in NCBI Gene Expression Omnibus (GEO; http://www.ncbi.nlm.nih.gov/geo; GSE76297).

## Abbreviations

BSA: Bovine serum albumin
CCA: Cholangiocarcinoma
eCCA: Extrahepatic cholangiocarcinoma
pCCA: Perihilar cholangiocarcinoma
iCCA: Intrahepatic cholangiocarcinoma
DAPI: 4′,6-Diamidino-2-phenylindole dihydrochloride
ECM: Extracellular matrix
MTT: 3-(4,5-Dimethyl-2-thiazolyl)-2,5-diphenyl-2H-tetrazolium bromide
TME: Tumor microenvironment
α: Alpha
β: Beta
shRNA: Short hairpin RNA
siRNA: Small interfering RNA
PBS: Phosphate buffered saline
P/S: Penicillin/streptomycin
FBS: Fetal bovine serum
