# Supplementary material for: Elevated ITGA2 expression promotes collagen type I-induced clonogenic growth of intrahepatic cholangiocarcinoma
Source: Sci Rep. 2022 Dec 27;12:22429. doi: 10.1038/s41598-022-26747-1 (PMC9794692; doi:10.1038/s41598-022-26747-1)
Supplement: Supplementary file 1 — Supplementary Figures. [file 41598_2022_26747_MOESM1_ESM.pdf]

Elevated *ITGA2* expression promotes collagen type I-induced clonogenic growth of intrahepatic cholangiocarcinoma

Chotirat Rattanasinchai, Panida Navasumrit and Mathuros Ruchirawat

Supplementary Information

(a) The list of differentially-expressed integrin adhesome in iCCA

#	Gene	Log_2FC (1.5, -1.5 cut off)	*p*-value (paired T test)	FDR-adjusted *p*-value
1	*ITGA2*	3.74	1.03E-45	1.19E-43
2	*ITGB8*	2.99	9.44E-38	1.82E-36
3	*FERMT1*	2.51	1.71E-34	1.88E-33
4	*TSPAN1*	2.37	3.27E-30	1.80E-29
5	*ITGA6*	2.36	1.71E-42	9.86E-41
6	*ITGB6*	2.34	1.07E-25	4.06E-25
7	*ASAP2*	2.14	2.11E-43	1.62E-41
8	*ITGB4*	2.14	3.15E-41	1.04E-39
9	*MMP14*	1.96	1.51E-41	5.80E-40
10	*TES*	1.92	9.18E-42	4.24E-40
11	*EZR*	1.85	2.35E-39	5.43E-38
12	*ITGAV*	1.79	1.77E-37	3.14E-36
13	*ITGA3*	1.65	2.03E-32	1.56E-31
14	*CAPN2*	1.54	2.30E-38	4.83E-37
15	*SORBS2*	-1.90	2.08E-31	1.76E-19
16	*MYOM1*	-1.90	7.08E-20	1.41E-30

(b)

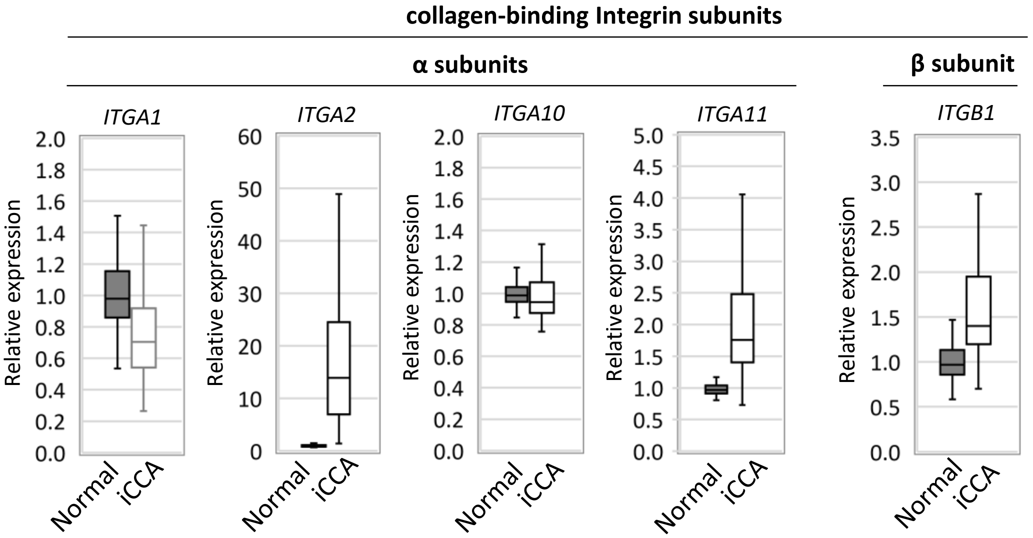

Supplementary figure 1 The list of differentially-expressed genes. (a) The list of top 16 differentially-expressed, integrin adhesome-related genes between intrahepatic cholangiocarcinoma (iCCA) tumors and their adjacent normal tissues from GSE76297 dataset (n = 91). **(b)** Box plot analysis of collagen I-binding integrin members from the same dataset.

(a)

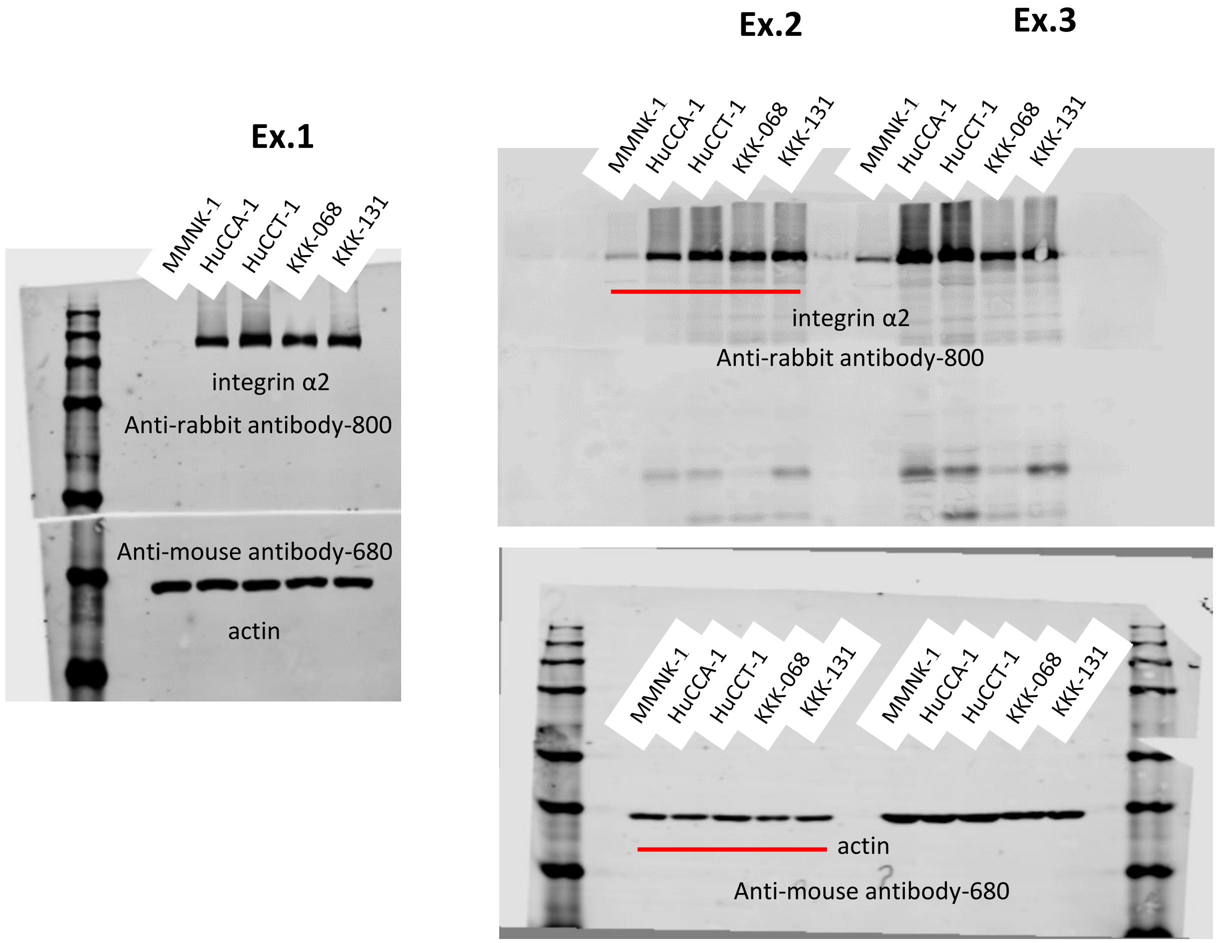

(b)

	Primary antibodies		Secondary antibodies	
	Type	Condition	Type	Condition
Top	Anti-integrin α2<br>(R)<br>(AB1936)	1:1,000<br>O/N, 4C	Goat-anti-mouse-800<br>Goat-anti-rabbit-680RD<br>(Li-Cor)	1:5,000 each<br>(mix)<br>1 h at RT
Bottom	Actin (M)<br>(sc-47778)	1:1000<br>O/N, 4C	Goat-anti-mouse-800<br>Goat-anti-rabbit-680RD<br>(Li-Cor)	1:5,000 each<br>(mix)<br>1 h at RT

Supplementary figure 2 Expression of integrin α2 in a cholangiocyte cell line and iCCA cell lines. (a) Immunoblot analysis of integrin α2 in MMNK-1, a cholangiocyte cell line, and intrahepatic cholangiocarcinoma cell lines: HuCCA-1, HuCCT-1, KKK-068, and KKK-131, from three independent experiments. Red lines indicate where the cropped images were obtained. **(b)** A table describes the antibodies and their concentrations used in this study.

(a)

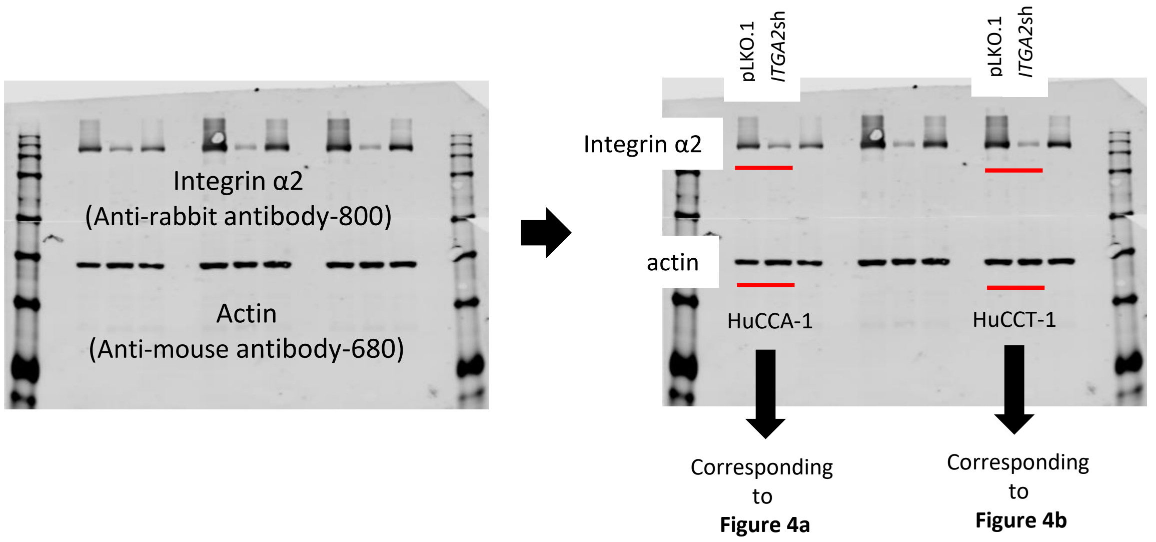

(b)

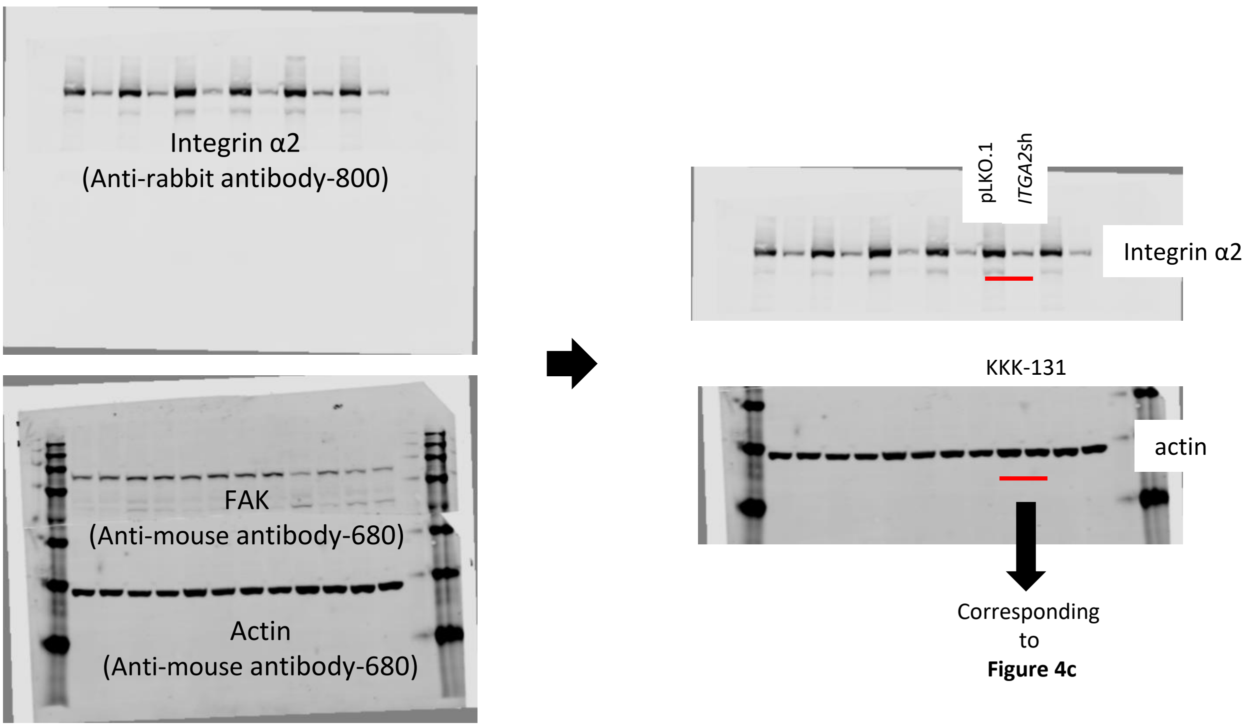

Supplementary figure 3 Uncropped blot images demonstrate the reduction of integrin α2 in *ITGA2*-stably silencing HuCCA-1, HuCCT-1, and KKK-131 cells, corresponding to Figure 4a-c. (a) Immunoblot analysis of integrin α2 and actin of HuCCA-1 and HuCCT-1, corresponding to **Figure 4a-b**. **(b)** Immunoblot analysis of integrin α2 and actin of KKK-131, corresponding to **Figure 4c**. Red lines indicate where the cropped images were obtained.

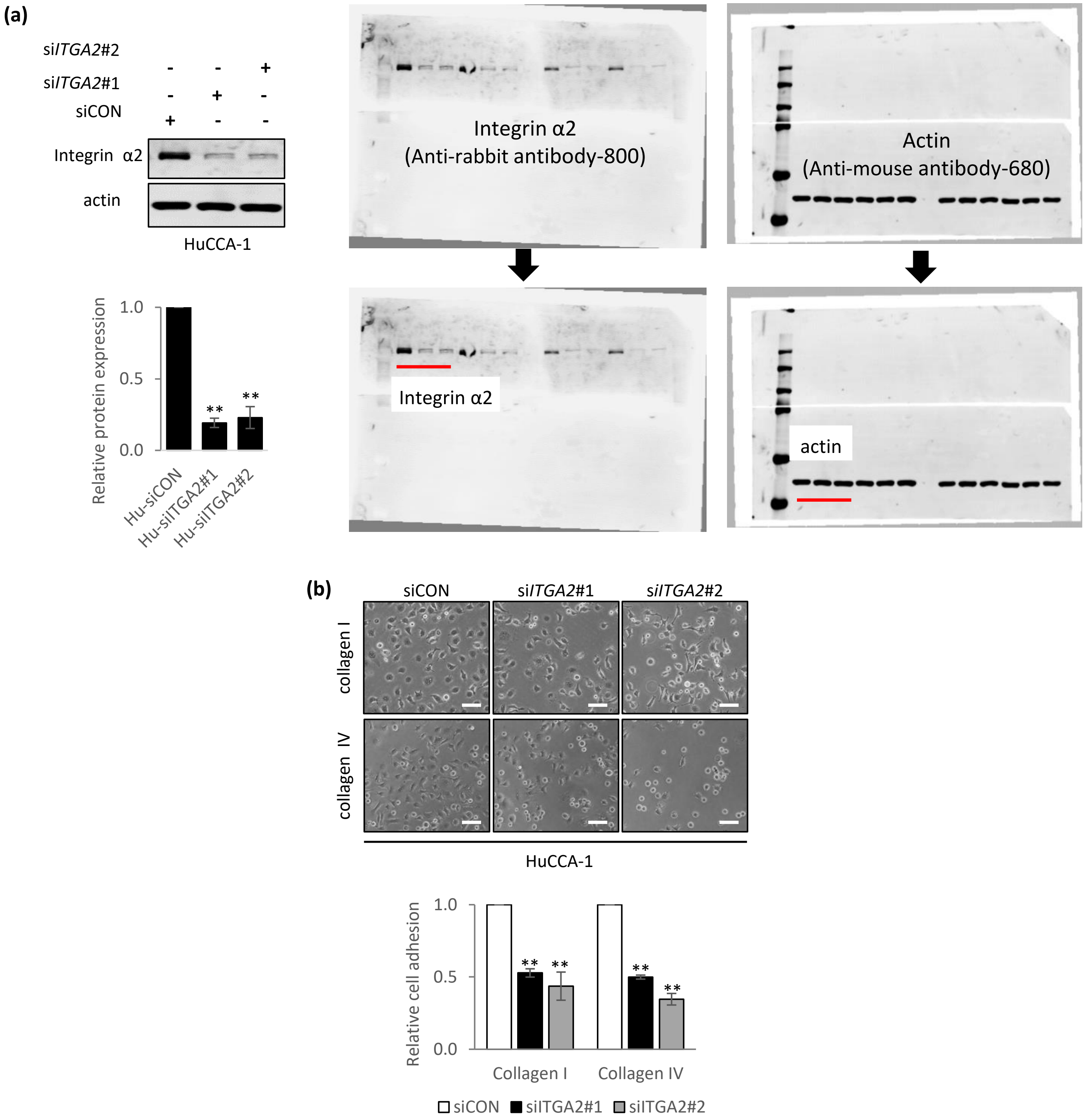

Supplementary figure 4 Transient silencing of integrin α2 decreases the ability of HuCCA-1 cells to adhere to collagen type I and IV. HuCCA-1 ± 100 nM control siRNA (siCON) or two different siRNAs targeting *ITGA2* gene (si*ITGA2*#1 and si*ITGA2*#2) for 40 h. **(a)** Immunoblot analysis against integrin α2. Actin serves as a loading control. **Right**: Original, uncropped blot images with red lines indicate where the cropped images were obtained, **Bottom**: Quantification of immunoblot analysis. Relative protein expression of each sample is normalized to its corresponding actin's band intensity. **(b)** Representative images from cell adhesion assay. Scale bar = 100 µm. **Bottom**: Quantification from at least three independent experiments. The bar graphs represent the mean ± SD from at least three independent experiments. The Student's t-test was used to calculate *p*-value, compared with a control group. ns, not significant; **, $p < 0.01$.

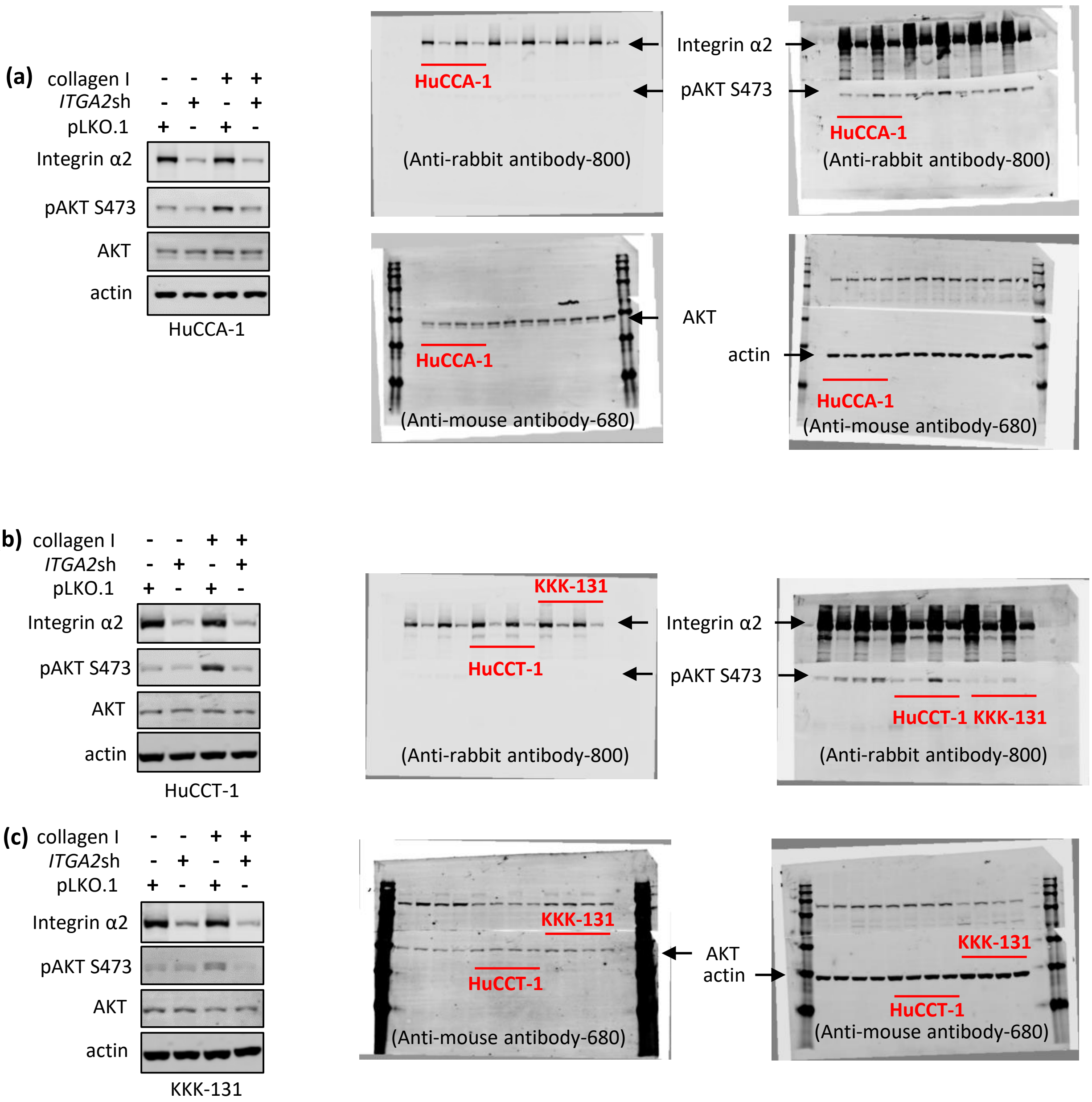

Supplementary figure 5 Uncropped blot images demonstrating that integrin α2 is required for collagen type I-mediated AKT phosphorylation, corresponding to Figure 5a-c. Immunoblot analysis of integrin α2, phosphor-AKT (serine 473), total AKT and actin of **(a)** HuCCA-1, **(b)** HuCCT-1 and **(c)** KKK-131 from **figure 5a-c** with their corresponding uncropped blot images shown on the right. Red lines indicate where the cropped images were obtained.

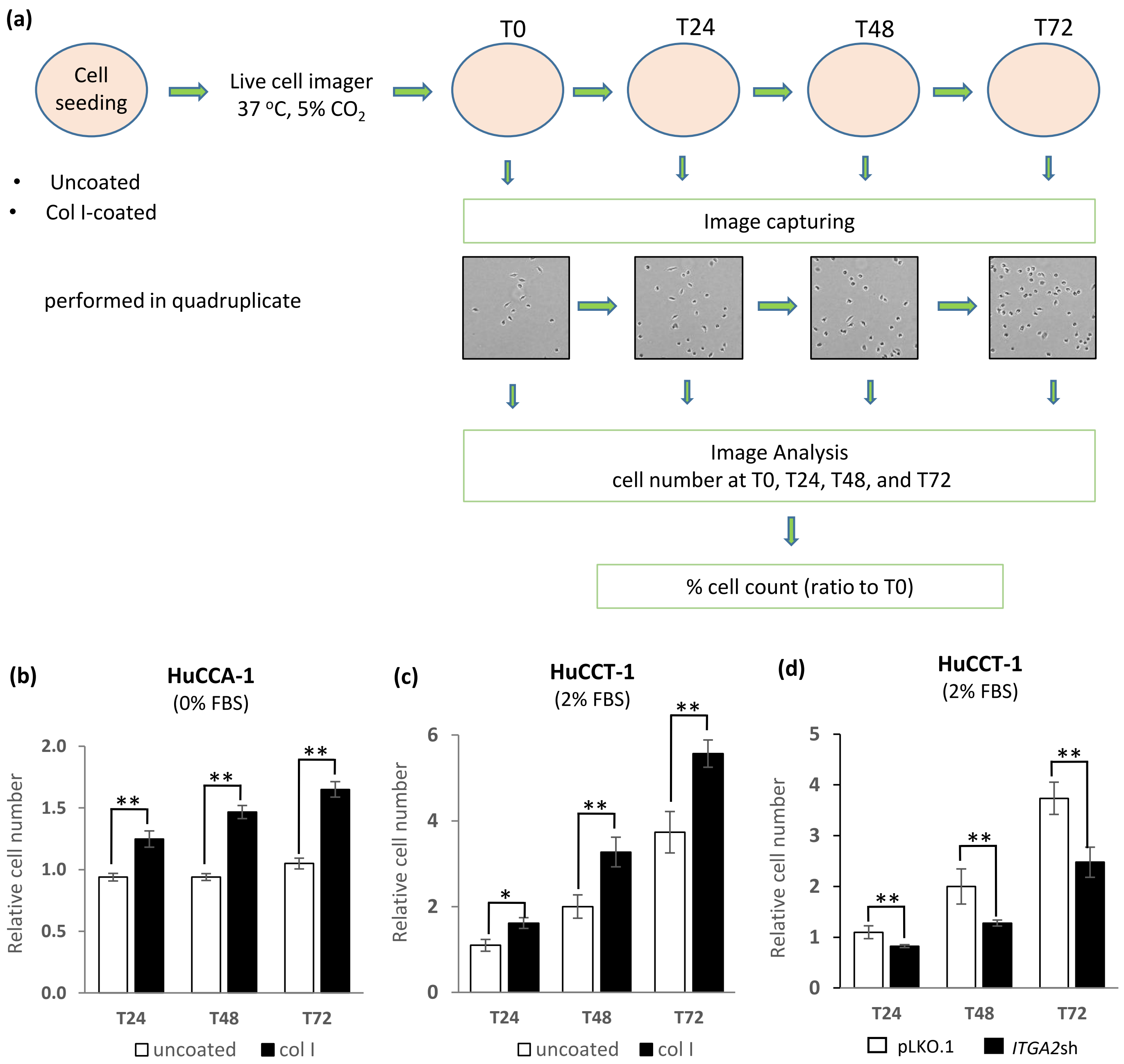

Supplementary figure 6 Collagen type I signals through integrin α2 to promote cell proliferation of iCCA cells. (a) A schematic diagram of the experimental procedure**. (b)** HuCCA-1 or **(c)** HuCCT-1 cells were seeded on the uncoated or collagen type I-coated, 96-well plates, **(d)** HuCCT-1 stably expressing pLKO.1 vector or pLKO.1-*ITGA2*sh constructs were seeded on the collagen type I-coated, 96-well plates. Cell proliferation was followed for up to 72 h, as judged by an increase in cell numbers within the individual wells. Bar graphs represent the results from three independent experiments performed in quadruplicates. *, $p < 0.05$; **, $p < 0.01$.
